# Segmental Colitis Associated with Diverticulosis: Status Quo

**DOI:** 10.3390/jcm15020646

**Published:** 2026-01-13

**Authors:** Gabriel Samasca, Dan Lucian Dumitrascu, Ciprian N. Silaghi, Iulia Lupan, Dinu Iuliu Dumitrascu

**Affiliations:** 1 Department of Immunology, University of Medicine and Pharmacy “Iuliu Hațieganu”, 400006 Cluj-Napoca, Romania; gabriel.samasca@umfcluj.ro; 2 2nd Medical Department, University of Medicine and Pharmacy “Iuliu Hațieganu”, Cluj Country Clinical Emergency Hospital, 400006 Cluj-Napoca, Romania; ddumitrascu@umfcluj.ro; 3 Department of Molecular Sciences, University of Medicine and Pharmacy “Iuliu Hațieganu”, 400349 Cluj-Napoca, Romania; silaghi.ciprian@umfcluj.ro; 4 Interdisciplinary Research Institute on Bio-Nano-Sciences, Babes Bolyai University, 400271 Cluj-Napoca, Romania; 5 Department of Anatomy, University of Medicine and Pharmacy “Iuliu Hațieganu”, 400304 Cluj-Napoca, Romania; dinu.dumitrascu@umfcluj.ro

**Keywords:** segmental colitis associated with diverticulosis, other colitidis, biologic therapy

## Abstract

The segmental colitis associated with diverticulosis (SCAD) is a particular presentation of the diverticular disease. Its effective management hinges on accurate diagnosis, prognosis, and ongoing therapeutic monitoring. Although SCAD generally follows a benign clinical trajectory, it remains the subject of intensive investigation. Recent advances in the field have yielded new opportunities to improve clinical outcomes for individuals with SCAD by informing diagnostic precision, risk stratification, and personalized treatment strategies.

## 1. Introduction

Diverticulosis is an anatomical alteration of the colon characterized by the herniation of mucosal and submucosal tissue through weaknesses in the muscularis propria. This process produces false diverticula and is frequently identified incidentally during imaging studies or endoscopic examination. The outward protrusion of mucosa and submucosa occurs at sites of wall weakness, often corresponding to penetrations by the vasa recta. Clinically, diverticulosis may be asymptomatic or present with episodic cramping and changes in bowel habits, and it constitutes a potential precursor to complications such as diverticulitis, bleeding, or perforation in some individuals [1]. In Western populations, diverticulosis affects more than 70% of individuals aged 65 years and older and represents the fifth-highest healthcare expenditure among gastrointestinal (GI) diseases in these regions [2]. Clinically, diverticulosis refers to the formation of multiple sac-like outpouchings (diverticula) along the GI tract, which can originate at sites of intrinsic wall weakness within either the small or large intestine. Notably, the condition predominantly affects the large intestine, with the sigmoid colon identified as the most commonly involved segment [3]. A paradigm shift has occurred in the classification and etiopathogenesis of diverticular disease (DD). Contemporary frameworks reconceptualize DD as a spectrum of clinically significant conditions rather than a single, uniform entity. This redefinition reflects advances in epidemiology, imaging, and pathophysiology and emphasizes the heterogeneity of presentation, progression, and response to treatment across patient populations [4]. Diverticulitis represents a heterogeneous clinical entity [5], and delineating the full spectrum of its presentations can enhance timely diagnosis and guide appropriate therapeutic decisions. The medical literature documents four uncommon variants of diverticulitis: non-sigmoid colonic diverticulitis, giant colonic diverticulum, segmental colitis associated with diverticulosis (SCAD), and small bowel diverticulitis. These atypical manifestations underscore the need for heightened clinical suspicion and careful radiologic and endoscopic assessment to distinguish DD from other inflammatory, infectious, or neoplastic processes [6]. SCAD remains a considerable challenge within contemporary research. Despite advances in endoscopic and histopathological assessment, the pathophysiology of SCAD remains incompletely understood, and its diagnostic criteria can overlap with other inflammatory processes of the colon. Current evidence suggests that SCAD is characterized by localized colonic inflammation adjacent to diverticula, without widespread involvement of the colon, which differentiates it from classic inflammatory bowel diseases (IBD). Management strategies are largely extrapolated from paradigms used for ulcerative colitis (UC) and other forms of colitis, with variability in therapeutic response and long-term outcomes. Ongoing investigations aim to clarify etiopathogenic mechanisms, optimize diagnostic algorithms, and establish standardized treatment protocols to improve symptom control, mucosal healing, and quality of life for affected patients [7]. Across the literature, several milestones have shaped the understanding of SCAD. In 1999, a study reported six patients diagnosed with DD accompanied by localized Crohn’s disease (CD) colitis, all of whom underwent segmental resection; the authors discussed the behavior and clinical significance of the co-occurrence of these two conditions within the same patient [8]. Between 2004 and 2006, reports on segmental colitis with diverticula characterized the condition as a localized disease [9,10]. In 2011, Tursi summarized the prevailing knowledge regarding this disease and proposed that SCAD constitutes a distinct and autonomous clinical entity [11]. However, clinical presentations of symptomatic uncomplicated DD may mimic IBD, whereas acute diverticulitis (AD) can be challenging to distinguish from SCAD [12]. Therefore, accurate differentiation of SCAD from other entities is of critical importance.

Building on the preceding analysis, this study addresses a central question: what is the current state of knowledge regarding the diagnosis, pathogenesis, biomarker landscape, clinical management, and prognosis of SCAD? The main objective was to synthesize existing evidence to delineate the contemporary understanding and to identify gaps for future research. Based on the evidence gathered, the secondary objective of our study was to present an integrated diagnostic algorithm that combines imaging, calprotectin, histology, and emerging biomarkers

## 2. Materials and Methods

By reviewing contemporary findings from PubMed database, we seek to synthesize current knowledge on epidemiology, pathophysiology, clinical presentation, diagnostic approaches, and therapeutic options related to SCAD, with the goal of identifying gaps in the literature and informing future research directions. PubMed is a free bibliographic database and search engine dedicated to literature in the biomedical and life sciences fields. PubMed’s administrative and technical headquarters are located in Bethesda, Maryland, United States. An initial PubMed search was conducted using the term “segmental colitis associated with diverticulosis” to identify the most significant and recent publications on the topic. This search identified 97 articles addressing SCAD. By applying prespecified inclusion criteria—studies with results strictly related to SCAD, including case reports—and exclusion criteria—studies whose conclusions referred to diseases associated with SCAD rather than SCAD itself—56 articles were eligible for analysis. It is important to note that DD-associated colitis may also be referred to as SCAD [13]. Additional studies [14,15] likewise demonstrate inconsistencies in terminology. Recognizing that reliance on a single keyword can risk omitting related terms and thereby limit comprehensiveness, a subsequent PubMed search employed multiple terms: “segmental colitis associated with diverticulosis,” “diverticular colitis,” “colitis associated with diverticulosis,” and “segmental colitis associated with diverticular disease.” The inclusion of these synonyms and related phrases enhances literature coverage and reduces the likelihood of missing pertinent studies. This expanded search identified 49 articles, all of which were included in the study.

## 3. Diagnosis of SCAD

The histopathological assessment of endoscopic GI biopsy specimens underpins the differential diagnoses of non-sigmoid colonic diverticulitis, giant colonic diverticulum, SCAD, and small bowel diverticulitis [16]. Within the context of DD, chronic active colitis is characterized as SCAD. This entity denotes persistent inflammatory changes confined to colonic segments bearing diverticula, often displaying mucosal and histologic features analogous to other IBD but restricted to diverticular-bearing segments. Endoscopic findings in SCAD range from mild erythema to ulceration, with histopathology demonstrating chronic active inflammatory infiltrates in the mucosa and submucosa, occasionally accompanied by crypt architectural distortion and lymphoplasmacytic infiltration. The condition highlights the heterogeneity of DD and the necessity for careful differential diagnosis to distinguish SCAD from UC and CD, given that management strategies and prognostic implications differ [14]. The pathogenesis of SCAD remains incompletely understood. Current evidence indicates a multifactorial process encompassing genetic predisposition, modifications in the gut microbiome, and instances of ischemia. Additional research is required to delineate the relative contributions and mechanistic interactions of these factors in SCAD etiology [15]. In published case series, SCAD has been reported in approximately 17% of cases, whereas segmental colitis without diverticulosis has been observed in as many as 50% of cases. These findings likely originate from studies investigating drug-induced colitis, specifically colitis associated with immune checkpoint inhibitors (ICIs) such as ipilimumab. The disparity between these percentages (17% vs. 50%) can be explained by the relationship between colonic diverticula and the anatomic distribution of inflammation induced by these therapies [17]. Population-based screening has identified subtle colonic anomalies consistent with SCAD in about 5% of individuals undergoing colonoscopy and in 12% of those with diverticulosis. SCAD appears to be associated with a lower incidence of adenomas and colorectal cancer compared with diverticulosis alone [18]. Pathophysiologically, SCAD is defined by an inflammatory response localized to the inter-diverticular mucosa, sparing the rectum and the right colon. In a cohort of 44 patients diagnosed with SCAD (30 males; median age 64.5 years; prevalence 1.99%, 95% CI 1.45–2.66%), individuals presenting with SCAD types D and B exhibited more pronounced symptomatology, elevated fecal calprotectin (FC) levels, a heightened requirement for corticosteroid therapy, and a diminished likelihood of achieving complete clinical remission. Although SCAD generally portends a benign clinical course, subtypes B and D have been associated with more severe symptomatology and a less favorable trajectory [19]. The patient population tended to be older, suggesting that SCAD may represent a subset of individuals predisposed to IBD who encounter an environmental trigger later in life [20].

### 3.1. Diagnostic Immunological Biomarkers

The presentation of leukocytosis in this context is described as infrequent, and standard microbiological assessments for enteric pathogens—including analyses of fecal bacteria and parasites—have repeatedly yielded negative results, offering no etiologic clarification. Serologic markers commonly associated with IBD, such as perinuclear anti-neutrophil cytoplasmic antibodies (pANCA) and anti-Saccharomyces cerevisiae antibodies (ASCA), are typically negative in SCAD. FC may aid in distinguishing SCAD from healthy controls or irritable bowel syndrome (IBS); however, it does not reliably differentiate SCAD from other IBD phenotypes [21].

**Figure 1:**
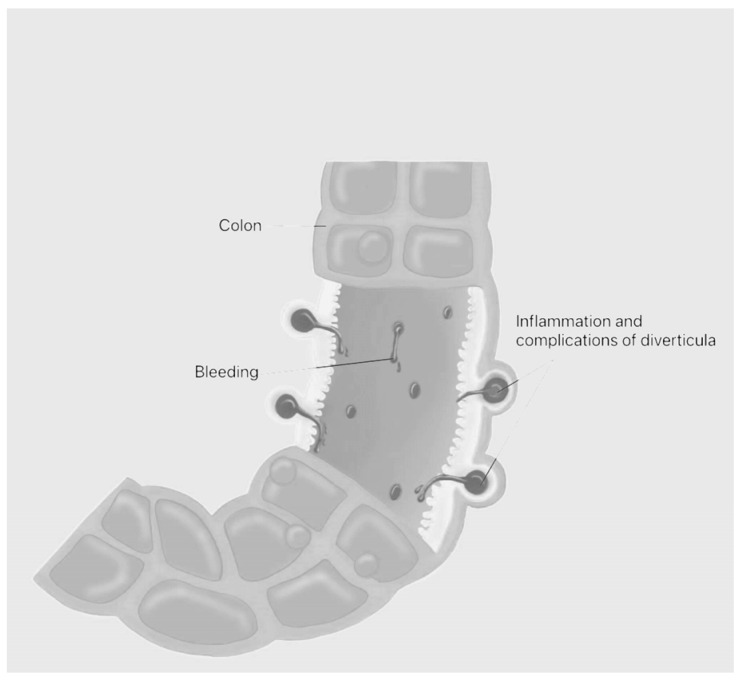
Colonic inflammation adjacent to diverticula. SCAD is defined by an inflammatory process that is confined to segments of the colon bearing diverticula, most commonly the sigmoid and descending colon, with relative sparing of the rectum and proximal colon. The hallmark inflammation pattern is segmental and patchy, corresponding to areas of DD rather than producing a continuous inflammatory distribution throughout the colon. This segmental pattern contrasts with the diffuse, continuous involvement typically seen in UC and with the transmural, often discontinuous lesions characteristic of CD.

Elevated tumor necrosis factor-alpha (TNF-α) expression appears to correlate with greater disease severity in DD, suggesting that TNF-α–mediated inflammatory pathways may contribute to DD pathogenesis and progression, aligning with the broader view of proinflammatory cytokines as biomarkers of disease activity [22,23]. Compared with IBS, TNF-α levels are significantly elevated in DD; in contrast, comparisons between DD subgroups (uncomplicated symptomatic and asymptomatic DD) or between symptomatic uncomplicated diverticular disease (SUDD) and controls show no significant mucosal differences, implying that TNF-α may contribute to pathogenesis in specific subgroups and could represent a therapeutic target for future interventions [24]. Additional case reports bolster the hypothesis that SCAD pathogenesis may be driven, at least in part, by TNF-α–mediated pathways, suggesting potential responsiveness to targeted TNF-α inhibitors or downstream signaling blockade. Collectively, accumulating evidence indicates that TNF-α–driven inflammation contributes to disease processes and raises the possibility that selective anti-TNF-α interventions might induce clinical and histopathologic improvement in a subset of SCAD patients [25]. Further literature documents instances in which SCAD emerged after bevacizumab therapy for metastatic colorectal carcinoma. These reports describe the temporal sequence from anti-angiogenic therapy initiation to localized segmental inflammatory changes confined to diverticular-bearing colonic segments, consistent with SCAD’s regional distribution and occurring without diffuse colitis or widespread architectural disruption. Endoscopic findings and histopathology reveal localized mucosal inflammation with inflammatory infiltrates and absence of granulomatous features. (Figure 1).

The clinical course emphasizes diagnostic, therapeutic, and prognostic considerations, highlighting the need for heightened clinical vigilance for SCAD in patients receiving bevacizumab who present with compatible symptoms and informing future management and research [26]. Case reports also describe a therapeutic response to adalimumab in a steroid-dependent SCAD patient, with resolution of symptoms and endoscopic/histologic improvement permitting corticosteroid tapering and withdrawal. These findings suggest that anti-TNFα blockade may be an effective treatment option beyond conventional corticosteroids and immunomodulators in steroid-dependent SCAD. Taken together, the literature supports the potential utility of monoclonal anti-TNF therapies, including infliximab and adalimumab, in the management of SCAD [27].

MicroRNAs (miRNAs) are known to target inflammatory pathways, including NF-κB signaling, which has also been implicated in SCAD etiology. Their non-invasive detection in stool is investigated, as fecal miR-21 expression levels are upregulated in IBD and able to drive colitis-related cancer, but its specific role in SCAD pathogenesis remains largely unknown [28]. Similarly to IBD, oxidative stress is part of SCAD’s inflammatory cascade. Superoxide dismutase (SOD) and neopterin could be SCAD diagnostic potential markers, as they indicate both oxidative damage and immune activation. Their SCAD and IBD specificity, however, are unknown and the clinical application is restricted due to the requirement for specialized assays [29].

This data integration evaluates key diagnostic features, pathogenic drivers, treatment implications, and emergent factors shaping future biomarker development. *(1) Negative Diagnostic Indicators.* A defining feature of SCAD is the absence of many systemic and serological markers commonly associated with infection or classic IBD. Systemically, leukocytosis is infrequent, and standard stool analyses for bacteria or parasites consistently yield negative results. Serologically, IBD-associated markers such as pANCA and ASCA are typically negative, making them useful rule-out tools to differentiate SCAD from UC or CD. FC often elevation in SCAD relative to IBS or healthy controls; however, FC lacks the specificity to distinguish SCAD from other IBD phenotypes, thus limiting its diagnostic discriminatory power. *(2) TNF-α as a Pathogenic Driver and Therapeutic Target.* Current literature emphasizes a framework for understanding SCAD through pro-inflammatory cytokines, with TNF-α positioned as a central driver. TNF-α expression is significantly elevated in SCAD and correlates with the degree of endoscopic mucosal damage, paralleling inflammatory patterns observed in IBD. Therapeutically, anti-TNF-α agents such as Infliximab and Adalimumab may be effective in severe or steroid-dependent cases, facilitating corticosteroid tapering and potentially inducing clinical remission. *(3) Iatrogenic and Emerging Factors.* Drug-induced SCAD has been reported in association with Bevacizumab, an anti-angiogenic therapy used for metastatic colorectal cancer, suggesting that vascular changes may trigger segmental inflammation in diverticular regions. Regarding future biomarkers, research is exploring miR-21 and oxidative stress markers (e.g., SOD, neopterin) as potential diagnostic tools. However, as of 2026, their clinical utility remains limited due to a lack of specificity and the requirement for specialized assays. The current understanding of SCAD highlights distinct diagnostic features, a cytokine-driven pathogenic framework with TNF-α as a key target, and emerging factors including iatrogenic associations and novel biomarker candidates. Continued validation and refinement of these markers are needed to enhance diagnostic precision and guide targeted therapy.

### 3.2. Radiological Diagnosis

Computed tomography (CT) continues to be regarded as the gold standard for the diagnosis of AD, due to its high sensitivity and specificity in detecting inflammatory changes of the colonic wall and surrounding tissues [30]. In the absence of clinical suspicion and without elevated tumor markers, the utility of CT in the routine surveillance of patients with GI malignancies remains uncertain [31]. In the Park et al. report, a case of SCAD is described in a 58-year-old male undergoing combination therapy with nivolumab and ipilimumab. A CT scan performed 17.9 weeks after initiation of immune checkpoint inhibitor therapy demonstrated diffuse wall thickening and mucosal hyperemia of the sigmoid colon, with concurrent engorgement of mesenteric vessels, findings concordant with a colitis pattern consistent with SCAD. The patient remained asymptomatic and was managed conservatively. A subsequent CT scan showed radiographic resolution of SCAD-related abnormalities in the sigmoid colon [32]. The study by Kim et al. reports a case involving a 63-year-old man who developed left lower quadrant pain and hematochezia during ipilimumab therapy. An axial CT image obtained at presentation demonstrated a SCAD pattern, defined by pronounced segmental and circumferential thickening of the sigmoid colon wall accompanied by pericolic fat stranding [33]. Nwankwo et al. report a case involving a 72-year-old male who presented with lower abdominal pain and hematochezia. Initial clinical assessment revealed nonspecific thickening of the large bowel, which raised concern for a malignant process. CT of the abdomen demonstrated thickening of the sigmoid colon with rectosigmoid involvement, thereby broadening the differential diagnosis to include both malignant and non-malignant etiologies [34]. Urquhart and colleagues conducted a retrospective analysis of seventy-five patients diagnosed with SCAD, of whom 48.0% were female, with a mean age at diagnosis of 62.5 years. Prior diverticulitis was documented in 37 patients (49.3%). The most common presenting symptoms were abdominal pain (33.3%) and hematochezia (22.7%). First-line therapeutic approaches most frequently employed were antibiotics (42.7%) and mesalamine (36.0%), while 20 patients (26.7%) required surgical intervention. The predominant initial endoscopic finding was isolated sigmoid inflammation in 86.7% of cases. Among 51 patients with histologically confirmed SCAD, 72 with diverticulitis, and 12 with CD, imaging studies were available for review. Penetrating disease was observed in 7 of 51 (13.7%) cases with SCAD, compared with 7 of 72 (9.7%) cases with diverticulitis and 2 of 12 (16.6%) cases with CD (*p* = 0.14). The authors conclude that SCAD should be considered when isolated sigmoid colon inflammation is identified on cross-sectional imaging and that penetrating disease is not a radiologic feature specific to SCAD or diverticulitis. We presented these data to elucidate the real-world profile of SCAD patients [35].

This synthesis examines how cross-sectional imaging, with a focus on CT, supports the identification of SCAD and outlines emerging clinical challenges tied to immunotherapy in 2026. CT remains the diagnostic cornerstone for AD, yet its application to SCAD reveals characteristic regional patterns that can mimic more serious diseases. The analysis also highlights the recognition of SCAD as a potential immune-related adverse event (irAE) associated with ICIs, and it summarizes demographic and management trends drawn from retrospective cohorts. Together, these insights underscore the need for careful radiologic and clinical interpretation to guide appropriate care. *1. CT Imaging: Diagnostic Gold Standard and Overlap*. CT is the established gold standard for AD, but its utility in SCAD is nuanced by distinct regional manifestations. Radiographic Signature: SCAD on CT typically shows circumferential wall thickening and mucosal hyperemia isolated to the sigmoid colon, frequently with mesenteric vessel engorgement, known as the vasa recta sign. Diagnostic Pitfalls: These findings can be non-specific and raise concern for malignancy or CD, particularly when rectosigmoid involvement is present. Penetrating Disease: Radiologic signs of penetrating disease (e.g., fistulas or deep tracts) are not unique to diverticulitis or CD and are observed in approximately 13.7% of SCAD cases, limiting their diagnostic utility for differential diagnosis. *2. Iatrogenic SCAD: The Immunotherapy Link.* A critical emerging trend is the recognition of SCAD as an irAE in oncology patients. Checkpoint inhibitors such as Nivolumab and Ipilimumab have been associated with SCAD patterns. Clinical Course: Iatrogenic SCAD may present with symptoms (e.g., hematochezia, pain) or be entirely asymptomatic and detected through routine surveillance CT imaging; in some instances, radiographic findings resolve spontaneously without aggressive intervention. *3. Clinical Demographics and Management Trends*. Patient Profile: Typical patients are in their early 60s, with nearly half having a prior history of diverticulitis. Primary Symptoms: Abdominal pain (33%) and hematochezia (23%) are among the most frequent presentations. Treatment Paradigm: First-line management commonly includes antibiotics (43%) and mesalamine (36%). Surgical Intervention: A significant minority (26.7%) require surgery, indicating that while SCAD is often benign, a subset of patients experiences recalcitrant or complicated disease. CT imaging remains essential for identifying the “isolated sigmoid inflammation” pattern characteristic of SCAD in 2026. Clinicians should maintain heightened vigilance for SCAD in patients receiving ICIs who present with colitis-like imaging, and they should not rely solely on the presence or absence of penetrating disease to exclude SCAD from the differential diagnosis.

### 3.3. Endoscopic Diagnosis

Colonoscopy plays a crucial role in establishing an accurate differential diagnosis among forms of chronic colitis that involve the colon with DD. In particular, it is instrumental in distinguishing CD from SCAD, as both conditions can present with colitis in diverticular-affected segments. Through endoscopic evaluation and targeted biopsies, colonoscopy aids in identifying characteristic mucosal appearances, distribution patterns, and histopathological features that differentiate inflammatory CD from SCAD, thereby guiding appropriate therapeutic strategies [36]. Diagnosis of SCAD is frequently impeded by non-ideal macroscopic descriptions of the colon and by limited biopsy sampling. A precise clinical assessment combined with endoscopic evaluation, together with an adequately designed biopsy sampling protocol and careful consideration of differential diagnoses, appears essential for prompt and accurate SCAD diagnosis [37]. In patients presenting with an initial endoscopic diagnosis of DD or SCAD, it is essential that current endoscopic classification schemes be systematically applied. The standardized use of these classifications yields predictive value for disease outcomes, enabling more accurate prognoses and informed clinical decision-making. Consistent application ensures uniform assessment across cases, facilitates comparability in research, and supports risk stratification that can guide therapeutic strategies and monitoring plans [38,39]. In cases of SCAD presenting with UC-like findings, a consideration should be given to aggressive anti-inflammatory therapy. This approach is warranted owing to the potential risks of severe ulceration, stenosis, and/or perforation [40]. In patients displaying endoscopic signs of erythema, friability, and ulcers—a presumptive diagnosis of SCAD was considered. To substantiate this preliminary assessment, multiple mucosal biopsy specimens were obtained for histopathological confirmation [41]. In the context of SCAD, lesions of the interdiverticular mucosa that exhibit diverticular and rectal sparing are not pathognomonic for SCAD. However, these histopathologic features may have prognostic relevance, potentially indicating disease presence. Accordingly, histological examination remains essential, and, when indicated, adjunct laboratory analyses should be employed to support an accurate SCAD diagnosis [42].

This synthesis argues for the central role of standardized endoscopy and biopsy protocols within the 2026 diagnostic framework for SCAD. It emphasizes that reliance on visual patterns alone is insufficient for definitive diagnosis. By mandating systematic endoscopic classification, strategic biopsy sampling, and integrated histopathologic interpretation, clinicians can improve diagnostic accuracy, risk stratification, and therapeutic decision-making, while distinguishing SCAD from CD and UC. *1. The Necessity of Systematic Endoscopic Classification.* The precision of SCAD diagnosis is frequently undermined by vague macroscopic descriptions. To mitigate this limitation, standardization is required: clinicians must systematically apply current endoscopic classification schemes (Types A–D). This standardized approach provides predictive value for disease outcomes, enabling better risk stratification and tailored monitoring plans. In addition, the “Rule of Sparing”—the observation that inter-diverticular mucosal inflammation with proximal and rectal sparing is the classic hallmark of SCAD—must be interpreted with caution. Although it remains a characteristic pattern, it is not pathognomonic; identical endoscopic appearances can occur in early CD, necessitating a high degree of clinical suspicion and corroborating evidence beyond visual assessment. *2. Strategic Biopsy Sampling Protocols*. Accurate differentiation between SCAD and IBD, specifically CD and UC, relies on a multimodal objective approach that begins at the vantage point of the endoscope. Targeted Biopsies should be obtained from multiple regions, including the inter-diverticular mucosa, the unaffected proximal colon, and the rectum. Histopathology remains essential but must be interpreted in conjunction with adjunct laboratory data to confirm SCAD and to exclude mimics. While features such as crypt architectural distortion and lymphoplasmacytic infiltration contribute to the diagnostic framework, their diagnostic weight is enhanced when integrated with clinical, laboratory, and radiologic findings within the 2026 framework. *3. Clinical Implications of UC-like Endoscopy (Subtypes B & D).* A critical subset of patients presents with endoscopic findings that resemble UC, corresponding to SCAD subtypes B and D. These cases warrant a more proactive management approach: aggressive anti-inflammatory therapy may be required to control disease activity and to mitigate complications. Risk mitigation is particularly important because patients in these subtypes are more susceptible to adverse outcomes, including stenosis, perforation, and chronic scarring. Early recognition and tailored intervention in this UC-like subset are essential components of optimal SCAD care within the 2026 diagnostic paradigm. Colonoscopy remains the instrumental tool for diagnosing SCAD; however, its diagnostic utility is maximized only through rigorous standardization of classification and biopsy protocols. Visual sparing of the rectum and diverticular orifices is not, in itself, a definitive diagnostic guarantee. Consequently, histopathology continues to serve as the gold standard for distinguishing SCAD from CD and for guiding the intensity of therapeutic strategies. The 2026 framework, therefore, relies on a harmonized approach that integrates endoscopic classification, targeted biopsy sampling, and histopathologic corroboration to achieve accurate diagnosis and appropriate management.

### 3.4. Histological Diagnosis

Histopathologic features typical of IBD can also be observed in SCAD, although the histopathology of UC frequently appears more severe [43]. In histological terms, SCAD exhibits feature that closely resemble those of chronic idiopathic IBD. This resemblance can complicate differential diagnosis, as the mucosal architecture, inflammatory cell infiltrates, and crypt architectural distortion may parallel patterns typically observed in CD or UC. Careful correlation with clinical presentation, radiographic findings, and endoscopic assessment is therefore essential to distinguish between diverticular-associated colitis and primary IBD, ensuring appropriate management [44]. In one case, a 73-year-old man presented with weight loss, abdominal pain, constipation, anemia, and several months of rectal bleeding. SCAD can be presented as a rare pseudotumor. This case highlights the diagnostic challenges posed by segmental colitis in the setting of DD, as the pseudo-tumoral form may mimic neoplastic, inflammatory, or infectious etiologies. Careful consideration of patient history, endoscopic findings, radiographic imaging, and histopathological examination is essential to distinguish this entity from more common differential diagnoses and to guide appropriate management [45]. In another case, a 57-year-old female with a history of colonic diverticulosis presented with chronic intermittent abdominal pain, non-bloody diarrhea, and hematochezia. Pathological examination revealed findings consistent with chronic colitis, including inflammation of the lamina propria, crypt distortion, and granuloma formation, which are characteristic of SCAD [46]. Ono and Gonzalez analyzed 81 cases of SCAD, with 79% involving the sigmoid colon, along with 166 IBD cases. An additional cohort of 27 patients had concomitant IBD and diverticulosis. In comparison with the IBD cohort, the SCAD cohort demonstrated a significantly lower frequency of several histologic features: crypt abscesses (20% vs. 45%, *p* < 0.0001), prominent basal lymphoid aggregates (37% vs. 51%, *p* = 0.042), crypt distortion (7% vs. 25%, *p* = 0.00090), Paneth cell metaplasia (37% vs. 57%, *p* = 0.0061), and crypt rupture (1% vs. 11%, *p* = 0.0089). Although these histologic features are not entirely specific, they may aid in differentiating IBD from SCAD, particularly when the clinical context is unclear or unavailable. The data are presented here because the observed quantitative differences facilitate differential diagnosis of SCAD [47]. Mastery of the most informative histological features, in conjunction with pertinent clinical data, is essential for routine clinical practice. This integrated expertise underpins precise pathological diagnosis and informs appropriate subsequent clinical management [48].

This synthesis delineates the histopathological nuances of SCAD as of 2026, with particular emphasis on its function as a diagnostic mimic of primary IBD and the quantitative distinctions that separate SCAD from UC and CD. It highlights the histologic features that complicate interpretation, the rare pseudo-tumoral presentation, and the critical role of integrated clinical–pathological correlation to minimize diagnostic errors. *1. The Histologic “Great Mimicker”.* SCAD frequently presents with a histological profile that parallels chronic idiopathic IBD, including crypt architectural distortion, lamina propria inflammation, and, in some cases, granuloma formation. A rare but clinically significant manifestation is the pseudo-tumoral variant, which can present with weight loss, anemia, and obstructive symptoms such as constipation. This variant risks misidentification as colonic neoplasia, deep infection, or severe CD. Given the overlap of features such as granulomas and cryptitis with IBD, pathology cannot be interpreted in isolation. Diagnosis requires strict correlation with the Sparing Rule (rectal and proximal colonic sparing) and radiographic evidence of diverticulosis. *2. Quantitative Differentiation: SCAD* vs. *IBD.* Relative to IBD, SCAD generally exhibits a lower severity of inflammatory markers: Crypt Abscesses: 20% in SCAD vs. 45% in IBD. Crypt Distortion: 7% in SCAD vs. 25% in IBD. Basal Lymphoid Aggregates: 37% in SCAD vs. 51% in IBD. Paneth Cell Metaplasia: 37% in SCAD vs. 57% in IBD. Crypt Rupture: Rare in SCAD (approximately 1%) vs. 11% in IBD, providing a strong negative predictor for SCAD. *3. Clinical–Pathological Integration.* Mastery of these subtle histological differences is essential for routine practice. The synthesis emphasizes that an integrated expertise—combining biopsy data with clinical history and endoscopic distribution—is the most reliable approach to avoid diagnostic errors. Comparative profile: SCAD vs. IBD. Inflammatory severity: Generally milder in SCAD; more severe in IBD. Crypt rupture: Very rare in SCAD (~1%); common in IBD (~11%). Granulomas: Possible and can mimic CD in SCAD; granulomas are common in CD. Anatomical limit: SCAD tends to be inter-diverticular with rectal/proximal sparing; IBD often shows different patterns of involvement. The pathology of SCAD represents a spectrum of chronic active colitis that is quantitatively less severe than IBD but qualitatively similar. The emergence of a pseudo-tumoral presentation underscores the necessity for multi-modal verification (imaging, endoscopy, and biopsy) to exclude malignancy. In this context, accurate diagnosis hinges on integrated clinical–pathological reasoning rather than isolated histology alone.

### 3.5. Differential Diagnosis of SCAD

SCAD is a distinct inflammatory colopathy characterized by segmental involvement of the colon, typically in diverticular-containing segments of the left colon. The differential diagnosis for SCAD is broad and includes infectious colitis, microscopic colitis (lymphocytic and collagenous types), ischemic colitis, eosinophilic colitis, autoimmune enterocolitis (including IBD with autoimmune features such as UC and CD), and segmental colitis without diverticulosis. Differentiation among these entities relies on integrating clinical presentation, endoscopic appearance, and histopathologic features. Endoscopic evaluation commonly serves as the entry point for differentiation. SCAD and segmental colitis related to diverticulosis typically show segmental, left-sided involvement near diverticula, with findings such as mild to moderate erythema, edema, and friability confined to affected segments. Infectious colitis may present with more diffuse or patchy mucosal injury, exudates, ulceration, or pseudo-membrane formation depending on the etiologic organism. Microscopic colitis, in contrast, generally preserves a normal gross mucosal appearance on colonoscopy, with microscopic inflammatory changes only evident on biopsy. Ischemic colitis characteristically exhibits pale, edematous, or violaceous mucosa with ulcers or hemorrhagic changes in watershed areas. Eosinophilic colitis frequently shows prominent eosinophilic inflammation in the mucosa or submucosa. Autoimmune enterocolitis may present with continuous involvement from the rectum (as in UC) or with skip lesions and transmural features (as in CD), alongside corresponding serologic and radiologic findings. Histological evaluation provides crucial, contributing differentiation. Infectious colitis is marked by neutrophilic cryptitis and crypt abscesses, with acute inflammatory changes and possible pathogen-specific organisms or toxins identified on ancillary testing. Microscopic colitis is defined by increased intraepithelial lymphocytes (lymphocytic type) or a thickened subepithelial collagen band with surface epithelial damage (collagenous type), often with minimal gross inflammation. Ischemic colitis shows mucosal and submucosal hemorrhage, with crypt architectural changes such as atrophy or drop-out, and may exhibit a lamina propria with edema and hyalinization. Eosinophilic colitis demonstrates prominent eosinophilic infiltration of the mucosa and/or submucosa, sometimes with peripheral eosinophilia. Autoimmune enterocolitis reveals chronic architectural distortion (crypt branching, dilation, and basal plasmacytosis) and may be associated with granulomas in CD or continuous mucosal involvement in UC. In SCAD, histology typically reveals segmental chronic inflammatory changes limited to diverticular-containing segments, with architectural distortion and inflammatory infiltrates that lack the pan-colonic acute features seen in infectious colitis or the transmural or granulomatous changes that characterize certain autoimmune enteropathies. Distinguishing SCAD from its mimics requires a coordinated assessment: endoscopy to define the distribution and surface patterns, and histology to characterize the dominant inflammatory pattern and architectural changes, all interpreted in the context of the patient’s clinical history and ancillary tests [16].

The differential diagnosis of SCAD versus IBD and diverticulosis is a common clinical challenge. Distinguishing these conditions relies on integrating clinical presentation with radiological, endoscopic, and histological findings. The following outlines how each modality can contribute to differentiation and what patterns are most informative. Radiological Evaluation. SCAD typically shows segmental colitis that correlates with areas of DD, most often in the sigmoid colon. The inflammation is usually localized to segments containing diverticula. Diverticulosis without inflammatory change may display diverticular outpouchings with little or no colonic wall thickening or pericolonic fat stranding. IBD patterns differ by type: UC often presents with continuous, left-sided or proctocolitis involving the rectum and extending proximally, while CD may show patchy, transmural inflammation with skip lesions, possible strictures, fistulas, and involvement beyond the colon (including the terminal ileum). CT or Magnetic Resonance (MR) colonography and cross-sectional imaging may reveal wall thickening, mucosal enhancement, and pericolic fat stranding. In SCAD, these findings are typically restricted to diverted segments; in IBD, they may be more diffuse or follow characteristic patterns (continuous in UC; transmural and segmental with skip areas in CD). Radiologic clues such as the presence of diverticula with localized inflammation and the absence of transmural fistulizing disease outside diverted segments support SCAD over CD; extensive bowel involvement or mural changes beyond diverticular regions favors IBD. Endoscopic Evaluation. SCAD endoscopy generally shows segmental mucosal inflammation confined to regions with diverticulosis. Findings include erythema, edema, friability, and superficial ulcers limited to those segments; the rectum is often spared. In diverticulitis with mucosal involvement, inflammation is likewise localized near diverticular colonic segments but may be less extensive if it represents simple diverticular inflammation without broader colitis. UC characteristically presents with continuous mucosal inflammation starting at the rectum and extending proximally, often with friability, granularity, and pseudo-polyps; CD shows skip lesions, patchy ulcers, cobblestoning, and may reveal fistulas or strictures in affected segments. Endoscopic appearance in SCAD helps distinguish it from IBD when the mucosal involvement strictly follows diverticular regions and lacks the continuity or transmural features seen in CD. Histological Evaluation. SCAD histology shows inflammatory changes limited to the mucosa and submucosa of diverticular segments. Findings may include chronic active colitis with neutrophilic infiltrates, crypt distortion, and basal plasmacytosis, but these changes are typically restricted to areas adjacent to diverticula and lack features that define the extent of disease beyond diverted segments. In UC, histology reveals continuous mucosal inflammation with crypt architectural distortion, goblet cell depletion, and inflammatory infiltrates; ulcers can be superficial or deep, and involvement is often continuous from the rectum. In CD, histology may show transmural inflammation with granulomas (when present), fissuring ulcers, and lymphoid aggregates; inflammation is often discontinuous and can involve any part of the GI tract. Diverticulitis-associated tissue changes include chronic diverticular inflammation with muscularis propria changes and fibrosis around diverticula; when isolated to diverticular segments without widespread mucosal pattern changes, this supports SCAD or diverticulitis rather than classic IBD. Integrated Diagnostic Approach. A thorough clinical assessment should consider age of onset, symptoms (diarrhea, blood, abdominal pain), pattern of bowel involvement, and extraintestinal manifestations. Correlate radiologic patterns with endoscopic and histologic findings: localized, diverticula-associated inflammation across imaging, endoscopy, and histology supports SCAD; continuous left-sided disease with rectal involvement suggests UC; patchy, transmural disease with possible skip lesions supports CD. In challenging cases, a multidisciplinary discussion and, if needed, serial assessments or targeted biopsies from representative segments (diverticular versus nondiverticular mucosa) can improve diagnostic accuracy. Noninvasive markers (e.g., FC) may aid in assessing inflammatory activity but are not definitive for differential diagnosis; they should be interpreted in the context of imaging, endoscopy, and histology. Distinguishing SCAD from IBD and diverticulosis hinges on the concordance of clinical features with segmental localization of inflammation on imaging, endoscopy, and histology. When inflammation remains strictly confined to diverticular segments with corresponding histologic changes and a sparing of nondiverticular mucosa, SCAD is favored. Diffuse, continuous inflammation starting at the rectum suggests UC, while skipped, transmural lesions with granulomas point toward CD. An integrated, multidisciplinary approach yields the most accurate differential diagnosis and informs appropriate management [30].

The differential diagnosis of SCAD requires careful differentiation from IBD and colorectal malignancy because management and prognosis differ substantially. Distinguishing SCAD from IBD and malignancy can be accomplished through an integrated diagnostic approach that emphasizes radiological, endoscopic, and histological evaluation. Radiological evaluation: Cross-sectional imaging and radiographic studies play a key role in characterizing the pattern and extent of colitis. CT or MRI enterocolonography and CT colonography can identify segmental bowel wall thickening that corresponds to regions of DD and may reveal a non-focal mass and preserved proximal segments. Features favoring SCAD include segmental involvement tightly confined to areas with diverticulosis, without the mass effect, transmural inflammation, or long contiguous involvement typical of CD. In contrast, imaging findings suggesting IBD may include diffuse or continuous colitis with skip lesions (as seen in CD) or diffuse, continuous rectal involvement (as typical of UC). Features suspicious for malignancy include focal masses, irregular stenosis, focal lymphadenopathy, and signs of invasion. Correlation with endoscopic findings and targeted biopsies is essential for accurate interpretation. Endoscopic evaluation: Colonoscopy allows direct visualization of the mucosal surface and assessment of the distribution of inflammation. SCAD characteristically shows segmental mucosal involvement that corresponds to diverticular segments, often with erythema, edema, friability, and superficial ulcers limited to diseased segments; normal-appearing mucosa may be present outside the affected regions. IBD typically exhibits more diffuse or continuous mucosal involvement (UC) or segmental, transmural-typical findings with potential fistulas and strictures (CD). Colorectal malignancy may present as an extrinsic impression, focal mass lesions, or irregular mucosal folds; biopsies are essential to exclude dysplasia or carcinoma. During endoscopy, obtaining multiple targeted biopsies from affected areas and from adjacent normal-appearing mucosa improves diagnostic accuracy and allows assessment of mucosal patterns that distinguish inflammatory from neoplastic processes. Histological evaluation: Biopsy specimens provide definitive tissue-based differentiation. SCAD on histology typically reveals nonspecific chronic inflammatory changes confined to mucosa and submucosa around diverticular sites, without the architectural distortion and basal plasmacytosis seen in UC, nor the transmural inflammation and granulomas associated with CD. Inflammatory changes may be localized and limited to diverticular-associated segments, with preservation of overall mucosal architecture outside the affected areas. IBD demonstrates characteristic features such as crypt architectural distortion, basal plasmacytosis, and, in CD, granulomas and transmural inflammation. Malignancy is suggested by dysplasia and malignant glands with invasion, requiring additional staging workup. A comprehensive panel of stains and, when indicated, immunohistochemical studies can aid in distinguishing inflammatory from neoplastic processes and in identifying any dysplastic changes within the colonic mucosa. Integrated diagnostic pathway: Given overlapping clinical presentations, a multidisciplinary approach—combining radiological, endoscopic, and histological data along with clinical context—is recommended. Correlation across modalities increases diagnostic confidence and informs appropriate management, which for SCAD typically centers on medical therapy and surveillance, whereas IBD and malignancy require disease-specific treatment strategies [34].

The differential diagnosis of SCAD requires careful discrimination from other common causes of lower GI symptoms, notably haematochezia, rectal bleeding, and diarrhoea. Accurate differentiation is essential because management strategies and prognostic implications differ markedly among these entities. This discussion outlines a structured approach to distinguishing SCAD from these conditions and describes the integral roles of clinical evaluation and endoscopic assessment in arriving at a timely and precise diagnosis. Clinical evaluation. History. Characterize the symptomatology: presence and nature of bleeding (bright red per rectum, melena, or occult blood), stool frequency, consistency, urgency, and the presence of mucus. Assess temporal pattern: abrupt onset versus insidious progression, intermittent vs. persistent symptoms, and episodes of acute hemorrhage. Elicit associated features: abdominal pain, tenesmus, weight loss, fever, night sweats, fatigue, and signs of anemia. Review risk factors and exposures: recent infections, antibiotic use, IBD, DD, hemorrhoids, colorectal neoplasia, coagulopathies, and use of antithrombotic agents. Past medical and surgical history: prior GI bleeding, abdominal surgeries, or known GI mucosal diseases. Physical examination. General assessment for signs of volume depletion or anemia. Abdominal examination for tenderness, guarding, or masses. Pelvic and rectal examination to assess for perianal sources (fissures, fistulas, hemorrhoids) and to gauge the presence of visible blood. Systemic review to identify extraintestinal manifestations suggestive of inflammatory or infectious processes. Differential considerations informed by history and exam. Haematochezia/Rectal bleeding due to anorectal sources (hemorrhoids, fissures, polyps, DD, colorectal neoplasia). Diarrhoeal disorders with secondary or associated bleeding (infectious colitis with mucosal bleeding, IBD with fragile mucosa, microscopic colitis with associated symptoms). Non-bleeding mimics or atypical presentations (functional diarrhoea, dietary intolerances, medication-induced changes) that may be misinterpreted as bleeding. Endoscopic evaluation. Indications and goals. Endoscopy is indicated for evaluation of suspected organic pathology causing bleeding or diarrhoeal symptoms and for direct mucosal assessment to identify sources such as ulcers, erosions, erosive colitis, inflammatory changes, masses, or vascular lesions. The primary goals are to localize a bleeding source, establish a definitive mucosal diagnosis where possible, obtain biopsies for histopathology, and guide therapeutic intervention when feasible Modality considerations. Colonoscopy (preferred for visible lower GI bleeding or suspected colitis/colorectal pathology): allows direct visualization, biopsy, and endoscopic therapy (e.g., hemostasis, polypectomy, dilation). Flexible sigmoidoscopy: may be sufficient when disease is suspected to be distal; limited examination of the proximal colon. Additional imaging/endoscopic techniques as adjuncts: capsule endoscopy or cross-sectional imaging (CT or MR enterography) when small bowel involvement is suspected; chromoendoscopy or magnification endoscopy to enhance mucosal detail where subtle inflammatory or neoplastic processes are suspected. Endoscopic findings and interpretation. Anorectal sources (e.g., hemorrhoids, fissures): identifiable lesions at the anorectal junction or anal canal with characteristic appearance; biopsy may be unnecessary if the source is clearly benign. Colitis and inflammatory conditions: mucosal edema, erythema, friability, longitudinal or transverse ulcers, pseudo-membranes, and friable mucosa; histology helps distinguish infectious, inflammatory, or ischemic etiologies. DD: diverticular orifice involvement with adjacent mucosal changes or diverticular bleeding sites. Neoplastic processes: polyps, masses, or suspicious mucosal irregularities requiring biopsy and staging workup. In cases where endoscopy is inconclusive or mucosa appears normal, consider additional investigations (stool studies for infection, laboratory tests for inflammatory markers or coagulopathy, imaging as indicated). Practical considerations. Preparation and timing: adequacy of bowel preparation, hemodynamic stability, and timing of endoscopy in the setting of active bleeding. Safety and contraindications: assessment for anticoagulation status, comorbidities, and patient tolerance for endoscopic procedures. Integration with multidisciplinary care: collaboration with gastroenterology, surgery, radiology, and pathology to interpret findings and plan management. Diagnostic algorithm. Initial assessment with focused history and physical examination. Laboratory investigations, including complete blood count (CBC), iron studies, inflammatory markers, and coagulation profiling, are commonly used to assess anemia, inflammatory status, and bleeding risk. First-line endoscopic evaluation (colonoscopy or sigmoidoscopy) to identify mucosal sources and obtain biopsies. If endoscopy is non-diagnostic or if small bowel disease is suspected, employ adjunctive imaging or specialized endoscopic techniques (capsule endoscopy, enterography, or targeted imaging studies). Synthesize clinical, endoscopic, histopathologic, and radiologic data to establish a differential diagnosis and guide management. Distinguishing SCAD from haematochezia, rectal bleeding, and diarrhoea hinges on a systematic approach that couples detailed clinical evaluation with targeted endoscopic assessment. The integration of patient history, examination, laboratory data, and direct mucosal visualization enables clinicians to localize pathology, characterize etiologies, and implement appropriate therapeutic strategies. Although this framework provides a structured approach, individual patient factors and local expertise may necessitate tailored pathways and interdisciplinary collaboration. A rigorous, stepwise approach to differential diagnosis—anchored in thorough clinical assessment and endoscopic evaluation—facilitates accurate distinction between SCAD and other common causes of rectal bleeding and diarrhoea. Clear documentation of presenting features, combined with decisive endoscopic findings and histopathology, underpins effective patient management and improves clinical outcomes [35].

The differential diagnosis of SCAD requires careful differentiation from several colonic diseases with overlapping clinical and endoscopic features. SCAD is characterized by inflammation confined to colonic segments with diverticulosis, most often in the left colon. Distinguishing SCAD from other colonic diseases relies on an integrated approach that combines endoscopic appearance, histopathology, clinical context, and ancillary testing. Differential diagnoses to consider—UC: typically presents with continuous colitis beginning at the rectum and extending proximally; may show mucosal friability and ulcers but lacks the segmental confinement to diverticulosis typical of SCAD. CD: can involve any part of the colon with skip lesions; may exhibit granulomas histologically and granulocytic inflammation with transmural involvement—features not confined to diverticular-bearing segments in SCAD. Infectious colitis: pathogens such as C. difficile, Salmonella, Shigella, Campylobacter, and parasites can mimic SCAD; endoscopy may reveal enlarging mucosal inflammation, exudates, or ulcerations, but stool studies and history of recent infection are essential. Ischemic colitis: often affects elderly patients with vascular risk factors; endoscopy may show pale, edematous mucosa with a sharp demarcation, usually in watershed areas; SCAD tends to be segmental and associated with diverticulosis rather than ischemia. NSAID (nonsteroidal anti-inflammatory drugs)-induced colopathy and drug-induced colitis: history of NSAID or other offending drug use; endoscopic findings can be patchy or diffuse, but distribution often correlates with drug exposure. Microscopic colitis (collagenous or lymphocytic): endoscopy is often normal or near-normal; histology reveals distinctive subepithelial changes or lymphocytic infiltration, which helps separate it from the mucosal inflammation seen in SCAD. DD without SCAD: diverticulosis with diverticular-related inflammation can occur outside the SCAD pattern and may lack the segmental mucosal changes tightly associated with diverticulosis seen in SCAD. How we do this endoscopically. Plan and perform colonoscopy with attention to segments containing diverticula, as SCAD inflammation is typically confined to these regions. Document the distribution of inflammation: assess whether involvement is segmental and limited to diverticular-bearing segments (supporting SCAD) versus continuous or patchy disease extending beyond diverticulosis. Obtain targeted biopsies from affected mucosa and adjacent normal-appearing mucosa, plus random biopsies from unaffected areas. Include samples for routine histology and, when indicated, additional stains. Correlate endoscopic findings with histology: SCAD often shows mucosal inflammation that is focal and limited to the diverticular segments; histology may show chronic inflammatory changes with acute inflammatory features confined to the mucosa and without the architectural distortion typical of long-standing UC. Integrate clinical data: assess symptom pattern (diarrhea, bleeding, abdominal pain), duration, recent infections, medication history (NSAIDs, antibiotics), systemic signs, and laboratory tests (inflammatory markers, stool cultures, *C. difficile* testing, FC when appropriate). Endoscopic features that help distinguish SCAD from mimics—SCAD: segmental left-sided colitis corresponding to diverticulosis, with erythema, edema, friability, shallow ulcers, or mild mucosal irregularity limited to affected segments; rectum spared or less involved; minimal crypt distortion; no granulomas. UC: continuous inflammatory involvement starting from the rectum, often with more uniform mucosal changes and crypt architectural distortion on histology. CD: focal or skip lesions with possible transmural involvement; granulomas may be seen histologically; any rectal involvement is variable. Ischemic colitis: abrupt mucosal changes in watershed areas; pale/ischemic-appearing mucosa; a distinct vascular pattern may be evident. Infectious colitis: acute inflammatory infiltrates with identifiable organisms or toxins; presence of organisms on testing and clinical history support infection. Microscopic colitis: normal endoscopy or subtle changes; histology shows collagen banding or lymphocytic infiltration without overt mucosal erosions typical of SCAD. An accurate diagnosis hinges on correlating endoscopic pattern with histologic findings and the clinical context. Endoscopic evaluation is central to identifying the segmental, diverticulosis-associated inflammation characteristic of SCAD, while histology and ancillary testing help exclude UC, CD, infection, ischemia, and drug- or microscopic-colitis mimics [36,37].

Distinguishing SCAD from IBD. Distribution and involvement: SCAD characteristically presents as segmental inflammation confined to segments with DD, often with sparing of non-diverticulosed mucosa. IBD, by contrast, commonly exhibits continuous (UC) or skip lesions with transmural or transmucosal involvement (CD) across broader segments or the entire colon. Endoscopic features: In SCAD, mucosal changes are typically localized to the sigmoid or left colon where diverticula are present. UC usually shows continuous colitis beginning at the rectum, while CD can show focal ulcers, fissures, and creeping fat, with a tendency for deeper, transmural involvement. Clinical context: SCAD often presents in patients with known diverticulosis and may have a different age distribution or symptom pattern compared with classic UC/CD phenotypes. Histology: The histological pattern is a pivotal discriminator (see “Histological evaluation” below). While overlaps exist, certain features favor SCAD or IBD when integrated with clinical and endoscopic data. Histological evaluation: how to do this and what to look for Biopsy strategy: Obtain multiple mucosal biopsies from affected segments with diverticulosis and from unaffected, normal-appearing mucosa for comparison. Include a range of sites if disease distribution is patchy, to assess for subtle changes elsewhere in the colon. Key histological features of SCAD: Inflammation confined to segments with diverticulosis, without widespread colonic involvement. Mixed inflammatory infiltrate in the lamina propria, with neutrophilic activity such as cryptitis or occasional crypt abscesses that may be present within the affected segment. Relative preservation of mucosal architecture in the most distal portions outside the diverticular segments; the crypt architecture in SCAD may be less distorted than in chronic UC. Absence of transmural inflammation, fissuring ulcers, and granulomas. Absence or limited presence of basal plasmacytosis and extensive architectural distortion characteristic of long-standing UC. Absence of granulomas (or only very focal, non-caseating granulomas) that would favor CD, though granulomas can be rare in both conditions and are not required for diagnosis. Key histological features that raise suspicion for IBD (to distinguish from SCAD): Continuous mucosal inflammation extending beyond diverticular segments, often starting at the rectum (UC) or showing transmural involvement and granulomas (CD). Marked crypt architectural distortion, basal plasmacytosis, and crypt apoptosis in chronic UC. Transmural inflammation, fissures, fistulae, or granulomas suggestive of CD. Ancillary considerations: Exclude infectious etiologies, ischemia, and drug-induced colitis that can mimic IBD or SCAD. Consider targeted or special stains and, when appropriate, molecular testing to exclude infectious or granulomatous etiologies. Correlate histologic findings with endoscopy, imaging, and clinical history to reach an integrated diagnosis. Integrated approach and implications. Distinguishing SCAD from IBD relies on an integrated approach that combines the distribution of disease, endoscopic appearance, and histological patterns. While histology provides essential clues, it is most informative when interpreted in the context of the patient’s DD distribution, symptomatology, and radiologic and endoscopic findings. Accurate differentiation informs management strategies, as SCAD is often treated differently from IBD, with consideration given to diverticular-related therapies and avoidance of unnecessary long-term immunosuppression typical for IBD in the absence of corroborating evidence [48].

The differential diagnosis of SCAD remains a clinical and pathological challenge because SCAD can mimic IBD and other forms of colitis. SCAD is a colitis that occurs in segments of colon already affected by diverticulosis, most often presenting in older adults with symptoms such as abdominal pain and changes in bowel habit. Distinguishing SCAD from uncomplicated diverticulosis or diverticular inflammation is crucial because therapeutic approaches and prognosis differ significantly. A careful integration of endoscopic findings, anatomical distribution, and histopathologic features guides accurate diagnosis and appropriate management. Endoscopic evaluation. Endoscopy is a central step in differentiating SCAD from diverticulosis alone. Colonoscopic examination typically reveals segmental or patchy mucosal inflammation that is confined to areas bearing diverticula, most commonly the sigmoid colon, with relative sparing of the rectum and proximal colon. Mucosal appearances can include erythema, edema, friability, shallow ulcers, or erosions within the inflamed segments, with preserved mucosa outside the involved areas. The presence of DD in the same region supports SCAD, but the pattern of involvement—segmental rather than continuous and distal-limited—helps distinguish SCAD from other forms of colitis. It is essential to obtain biopsies from both involved and uninvolved mucosa to characterize the inflammatory pattern and to exclude concomitant pathologies. Histological evaluation. Histology provides critical corroboration for the endoscopic impression. Biopsies from affected segments typically show mucosal inflammation with neutrophilic infiltration, cryptitis, and sometimes crypt abscesses. The lamina propria may contain chronic inflammatory cells, and there can be varying degrees of crypt architectural distortion consistent with chronic inflammation. Importantly, features such as granulomas (which would suggest CD) are generally absent or non-diagnostic in SCAD. The histological pattern may resemble IBD but remains restricted to segments associated with diverticulosis. A comparison with biopsies from normal-appearing mucosa elsewhere in the colon aids in reinforcing the segmental nature of the inflammation. Correlation with clinical presentation, endoscopic distribution, and absence of transmural involvement supports the diagnosis of SCAD rather than other etiologies. How do we do this? A systematic approach combines clinical assessment with targeted endoscopic and histological evaluation. Clinicians should: Assess symptomatology and exclude alternative explanations such as infectious colitis or ischemic processes. Perform colonoscopy with careful documentation of the inflammatory distribution, focusing on segments around diverticula and noting any rectal involvement. Obtain adequate biopsies from inflamed and non-inflamed mucosa for histopathologic analysis, ensuring samples reflect both the affected region and surrounding normal-appearing mucosa. Integrate endoscopic and histological findings with clinical context to distinguish SCAD from diverticulosis alone, DD with diverticulitis, UC, CD, or ischemic colitis. Distinguishing SCAD from diverticulosis and related colitides hinges on recognizing the segmental, diverticula-associated pattern of inflammation and corroborating it with endoscopic and histological evidence. A disciplined, integrated diagnostic approach minimizes misclassification and informs appropriate therapeutic strategies, ultimately guiding better patient outcomes [49,50].

### 3.6. An Integrated Diagnostic Algorithm Combining Imaging, Calprotectin, Histology, and Emerging Biomarkers

#### 3.6.1. Weighed of Conflicting Evidence Common in Presented SCAD Studies

The primary conflicting evidence regarding SCAD involves the following:

- the presence and distribution of inflammatory markers across different disease states;
- the presence of ambiguities in diagnostic imaging. While CT is considered the gold standard for AD, its utility for SCAD is uncertain, with findings sometimes showing a clear pattern and at other times being nonspecific and raising suspicion for malignancy. Penetrating disease is not a specific radiologic feature for SCAD, diverticulitis, or CD. Furthermore, CT findings consistent with SCAD can be present in both asymptomatic and symptomatic patients. More information is available from various medical reports;
- the diagnostic specificity of its defining endoscopic and histopathological features;
- histopathologic features of SCAD can closely resemble or be analogous to chronic idiopathic IBD, making differentiation challenging. However, a large analysis comparing SCAD and IBD cases found statistically significant differences in the frequency of features like crypt abscesses and crypt distortion, which may aid in distinguishing the conditions.

The conclusion of the analysis of conflicting evidence is that an integrated diagnostic algorithm combining imaging, calprotectin, histology, and emerging biomarkers is absolutely necessary in medical practice.

#### 3.6.2. The Diagnostic Algorithm for SCAD

The approach unfolds through four sequential steps; each aimed at maximizing diagnostic accuracy while guiding appropriate management.

*Step 1: Clinical Suspicion and Initial Laboratory Evaluation.* Presenting symptoms typically include chronic diarrhea, cramping in the left lower quadrant, and intermittent hematochezia in an older patient (generally aged 60 or above) with known diverticulosis. Initial laboratory workup focuses on ruling out infection, using stool cultures or PCR. A CBC is usually normal, and fever and leukocytosis are characteristically absent, which helps distinguish SCAD from AD.

*Step 2: Non-Invasive Biomarkers and Imaging*. FC levels are typically elevated in SCAD, reflecting mucosal inflammation and aiding differentiation from IBS or healthy controls. However, FC cannot reliably distinguish SCAD from IBD. Emerging serological biomarkers, such as pANCA and ASCA, are generally negative in SCAD and can assist in ruling out UC or CD. Imaging via CT or ultrasound may show long-segment circumferential wall thickening involving the sigmoid/left colon with engorged mesenteric vessels (vasa recta). A key differentiator is that SCAD demonstrates inflammation between diverticula but lacks significant pericolonic fat stranding or abscesses typical of AD; SCAD also tends to spare the rectum and terminal ileum.

*Step 3: Endoscopic Assessment*. Endoscopy reveals inflammation localized strictly to the inter-diverticular mucosa. The recommended procedure is a full colonoscopy to confirm rectal sparing and the absence of proximal colonic involvement.

*Step 4: Histopathology*. Biopsies should be obtained from the affected segment as well as the proximal colon and rectum to confirm the diagnosis. Histopathologic findings typically show chronic active colitis with mild crypt distortion and focal cryptitis localized to the diverticular segment. Granulomas are generally absent, and there is a lack of transmural inflammation or fistula formation, which helps to rule out CD.

## 4. Dietary Management

In this context, dietary restrictions do not appear to prevent the recurrence of diverticulitis. Current evidence suggests that while certain dietary measures—such as a high-fiber diet—may reduce symptoms and potentially lower the risk of future diverticular complications in some patients, they do not reliably avert recurrent episodes for all individuals. Consequently, management of recurrent diverticulitis should be individualized, taking into account the patient’s clinical history, comorbidities, and overall risk profile, and may involve a combination of dietary modification, medical therapy, and, when indicated, surgical consultation [51]. The frequency of episodes appears to be associated with the adoption of low-fiber dietary patterns, as well as with prolonged colonic transit time and elevated intraluminal pressure resulting from low-volume stools [52]. A concomitant low-fiber diet combined with a high intake of red meat and saturated fat may constitute a risk factor for diverticulitis. This dietary pattern could contribute to increased intraluminal pressure and altered colonic motility, potentially promoting diverticular inflammation when diverticula are present [53]. Piotrowicz and colleagues analyzed dietary components and found that individuals in the SUDD cohort exhibited higher fat intake and lower vitamin E consumption, particularly among those with SCAD. Furthermore, across all analyzed groups, there was a pronounced reduction in the intake of calcium, magnesium, and zinc, accompanied by an approximately one-third decrease in dietary fiber intake. Based on these nutritional data, several factors may contribute to the development of DD, including high consumption of animal protein, fat, and cholesterol; micronutrient deficiencies (notably zinc); excessive sodium intake; and the excessive consumption of B vitamins, especially vitamin B6 [54]. The available data, though not definitive, indicate that a majority of patients respond favorably to treatment incorporating a high-fiber diet, antibiotics, and/or 5-aminosalicylic acids (5-ASA) [55]. In many cases of SCAD, resolution is achieved through the combined use of a high-fiber diet and antibiotics. Salicylates are typically reserved for more severe presentations of the condition [56].

## 5. Pharmacological Management

SCAD represents a distinct inflammatory condition localized to the colonic segments that harbor diverticula, without evidence of widespread colitis. The therapeutic approach to SCAD encompasses both pharmacological and non-pharmacological strategies, tailored to disease activity and symptom severity (Table 1).

More recently, biologic therapies that target specific inflammatory pathways, such as anti-TNF agents, have been explored in cases of refractory SCAD. Given the heterogeneity of this condition, treatment should be individualized to the patient’s clinical circumstances. However, the current evidence base remains limited, underscoring the need for further rigorous studies to define optimal therapeutic strategies and to assess long-term safety and efficacy.

SCAD is a distinct inflammatory colopathy characterized by segmental involvement of the colon in regions bearing diverticula. It presents diagnostic and therapeutic challenges for colorectal surgeons, given its clinical overlap with UC and CD and its implications for surgical decision-making. This overview outlines the defining features of SCAD, the diagnostic approach to differentiate it from other IBD, and the management considerations that influence surgical planning and outcomes. SCAD refers to inflammatory changes confined to colon segments with diverticulosis, most often involving the sigmoid colon. Unlike diffuse colitis, the inflammation in SCAD is segmental and can spare uninvolved mucosa. The condition typically affects adults, frequently in the older population, and may present with chronic diarrhea, left lower quadrant pain, and rectal bleeding. Because its endoscopic and histological appearance can resemble UC or CD, accurate differentiation is essential to avoid inappropriate treatment pathways. Diagnostic approach. A systematic diagnostic workflow is essential for accurate identification of SCAD. Colonoscopy with targeted biopsies from inflamed and non-inflamed segments, in conjunction with radiologic imaging when indicated, helps delineate the extent and distribution of disease. Histopathology often shows chronic inflammatory changes without granulomas, and findings are localized to diverticular-bearing segments. Important differential considerations include UC, CD, infectious enterocolitis, and ischemic colitis. A multidisciplinary evaluation involving colorectal surgeons, gastroenterologists, and pathologists improves diagnostic precision and guides management. Management and surgical implications. Medical therapy for SCAD frequently involves anti-inflammatory strategies (for example, 5-ASA) and, in some cases, antibiotic regimens. Importantly, many patients respond to medical management, and unnecessary colectomies can be avoided with correct diagnosis. Colorectal surgeons are integral when surgical intervention becomes necessary—such as in cases of complicated DD, luminal obstruction, perforation, or refractory inflammation. Surgical decision-making should consider the segmental nature of SCAD, preserve unaffected colon when possible, and address comorbid DD or complications. Postoperative outcomes are generally favorable when SCAD is correctly identified and managed within a multidisciplinary framework. Awareness of SCAD as a distinct, segmental inflammatory process associated with diverticulosis is crucial for colorectal surgeons. Accurate differentiation from other forms of IBD informs appropriate medical management and prevents unnecessary surgical procedures. A collaborative, multidisciplinary approach enhances diagnostic accuracy, optimizes treatment strategies, and improves patient outcomes in SCAD.

## 6. Prognosis

The researchers requested the adoption of appropriate preventive strategies to mitigate DD [64]. SCAD represents a relatively novel and emerging clinical entity. In the current literature, the majority of patients diagnosed with diverticular associated colitis (DAC) and SCAD experience a benign clinical course [65,66]. When compared with IBD, SCAD demonstrates a comparatively more favorable prognosis, as evidenced by a lower incidence of complications [67]. Given the differences in natural history and prognosis between SCAD and IBD, long-term pharmacotherapy is frequently unnecessary in SCAD [68]. In the majority of cases, SCAD responds favorably to medical therapy [69]. In severe presentations, SCAD may possess the potential to progress to UC. UC is characterized by an often-aggressive disease course that can necessitate colectomy in affected individuals [70]. Atypical manifestations of SCAD have been documented in the literature. While the classical presentation includes localized inflammation around diverticula with left-sided or sigmoid involvement, several reports describe non-classical or atypical patterns, such as isolated colonic segments outside the sigmoid colon, segmental non-necrotizing inflammation, or presentations that mimic other colitides. These atypical cases underscore the heterogeneity of the disease and highlight the importance of considering SCAD in the differential diagnosis when patients present with compatible histopathology and imaging findings but unusual clinical or anatomical distribution. In a representative case, a patient presenting with marked inflammatory changes, including enterocolonic fistulae and hydroureteronephrosis, underscores the likelihood of substantial segmental colitis associated with complications related to diverticulosis [71]. Surgical intervention is indicated for patients with recurrent diverticulitis or its complications, such as peridiverticular abscess, perforation, fistulizing disease, strictures, or obstruction [72]. In addition, a rare postoperative complication—pyoderma gangrenosum—has been described in association with segmental colitis linked to diverticulosis [73].

## 7. Limitations of the Study

### 7.1. Fragility of Therapeutic Recommendations

The manuscript suggests that anti-TNF therapies “could improve outcomes,” yet this assertion rests on an extremely thin evidence base. The primary evidence comprises isolated case reports. This represents a critical limitation because more than 80% of SCAD cases resolve spontaneously or respond to only mild 5-ASA treatment. In the absence of RCTs, it is impossible to determine whether the reported success of biologics reflects the intervention itself or the natural benign course of the disease.

### 7.2. Implementation Barriers for Biomarkers

Although emerging biomarkers are described as “promising,” the manuscript presents a substantial practical contradiction. The clinical gap is that these assays are expensive and not standardized. As of 2026, the lack of standardized definitions and validated reference values remains a primary barrier to translating these tools from research settings into routine clinical use. Additionally, many inflammatory biomarkers are non-specific and may reflect general mucosal irritation rather than providing a diagnosis specific to SCAD.

### 7.3. Ambiguity in Disease Progression

The manuscript notes that SCAD may “progress” to UC, but it omits critical contextual details. Undefined proportions are reported in the literature; only a small subset of patients—approximately 2.3% in some prospective cohorts—develop UC following a SCAD diagnosis. There is a major debate over whether these cases represent true biological progression or are simply early-stage IBD misclassified as SCAD at initial presentation. The manuscript’s failure to address this distinction leaves a gap in understanding the true long-term prognosis.

### 7.4. Overlooked Demographic and Diagnostic Limitations

Atypical presentations are acknowledged but not quantified in terms of diagnostic delay risk. In older populations, SCAD is frequently misdiagnosed as ischemic colitis or diverticulitis, potentially leading to inappropriate surgical interventions. Regarding imaging, the manuscript advocates radiologic approaches; however, 2026 data indicate that non-invasive tests such as CT coronary angiogram have limited sensitivity (as low as 72%) for identifying smaller unhealed lesions, which can contribute to under-diagnosis.

### 7.5. Methodological Limitations of Older Studies in Relation to Modern SCAD Classification Schemes

Early investigations often relied on retrospective chart reviews, small case series, and referral-center cohorts, which introduce substantial selection bias and limit generalizability. Definitions for SCAD were frequently inconsistent or ill-defined, with variable distinctions made between SCAD, CD, UC, diverticulitis, and other inflammatory or infectious colitides, leading to frequent misclassification. In many cases, diagnostic criteria depended on non-standardized endoscopic impressions or radiologic assessments rather than unified, reproducible criteria, compromising reproducibility across studies and centers.

Together, these limitations underscore the need for prospective, controlled studies, standardized biomarker definitions, and careful differentiation of disease trajectories to more accurately define prognosis and inform treatment strategies.

## 8. Conclusions

SCAD is an emerging entity with, on the whole, a favorable prognosis relative to IBD, although attention to its atypical presentations and potential complications remains essential for accurate diagnosis and appropriate management.

### 8.1. Analysis of Tensions in Conclusions

Five dominant tensions emerge across the domains of therapeutic strategy, biomarker development, diagnostic precision, dietary guidance, and prognostic framing. *Therapeutic Potential* vs. *Evidence Deficit*: Substantial tension exists between the pathogenic evidence supporting TNF-α–driven inflammation and the clinical readiness to deploy anti-TNF-α biologics. Although the discussion suggests that anti-TNF-α therapies could improve patient outcomes, this optimism is tempered by the need for prospective studies to define safety profiles and appropriate patient selection. The resulting management dilemma arises from a scientific rationale pointing toward a specific therapeutic solution, yet insufficient data prevent its adoption as a standard frontline treatment. *Biomarker Innovation* vs. *Clinical Utility*: A second major tension arises between technological promise and practical accessibility. Emerging biomarkers (e.g., miRNAs and microbiome signatures) offer high diagnostic potential, yet they currently fail the routine adoption test due to gaps in standardization, validation, and broader accessibility. Consequently, clinicians may rely more on traditional, invasive, multidisciplinary frameworks (radiology, endoscopy, histopathology) rather than simplified molecular testing. *Diagnostic Complexity* vs. *Management Urgency*: A third tension persists between the need for precise differentiation from IBD and the integrated, multidisciplinary effort required to achieve it. Because SCAD mimics other colitides, the literature advocates for a structured diagnostic framework. The risk of misdiagnosis remains high, while the resources and processes necessary for precise discrimination are substantial and resource-intensive. *Dietary Generalization* vs. *Individualized Care*: The discussion identifies a conflict in dietary management for DD. Although high-fiber diets are broadly recognized for symptom relief, there is a paucity of robust long-term guidelines. This creates tension between endorsing wide-ranging dietary changes and the need for a nuanced, individualized approach that current prospective research cannot yet fully support. *Prognostic Optimism* vs. *Atypical Risks*: Finally, there is a tension between the generally favorable prognosis attributed to SCAD relative to IBD and the potential for atypical presentations and complications. Clinicians must balance a generally positive outlook with vigilant attention to rare but severe outcomes that could be overlooked given the disease’s overall mild reputation. Collectively, these tensions highlight the need for careful interpretation of evolving evidence, targeted prospective research, and the integration of therapeutic, biomarker, diagnostic, and dietary considerations within multidisciplinary, patient-centered care pathways.

### 8.2. Established Clinical Knowledge

The following components are prioritized in contemporary practice and guide clinical decision-making. *Multidisciplinary diagnostic approach*: Accurate identification of SCAD requires the integration of radiology, endoscopy, and histopathology. This triad is regarded as the most reliable method to differentiate SCAD from IBD. *Stepwise pharmacotherapy*: Management follows a validated escalation pathway based on disease severity. Mild disease is typically addressed with 5-ASA; moderate disease with locally acting corticosteroids (e.g., budesonide); and chronic or steroid-dependent disease with immunomodulators (e.g., thiopurines). *Prognostic outlook*: It is generally recognized that SCAD carries a more favorable prognosis than IBD, with a lower risk of systemic complications and a higher likelihood of spontaneous remission or treatment-induced resolution. Basic dietary support: High-fiber diets are acknowledged for providing symptomatic relief and partial risk reduction within the context of DD. These core elements guide contemporary clinical practice and underscore the emphasis on accurate diagnosis, staged therapy, a prognosis more favorable than that of IBD, and supportive dietary considerations.

### 8.3. Preliminary Hypotheses & Research Frontiers

These elements represent the “cutting edge” but remain speculative due to a lack of robust, large-scale data: *TNF-α Pathways as Therapeutic Targets*: While it is hypothesized that TNF-α drives SCAD pathogenesis, the use of biologics (anti-TNF-α) is currently a hypothesis-driven “possibility” for refractory cases. It lacks the prospective efficacy and safety data required for standard labeling. *Novel Biomarkers*: The use of miRNAs, oxidative stress mediators, and microbiome signatures remains a preliminary hypothesis for non-invasive monitoring. These are currently restricted by high costs, lack of standardized reference ranges, and poor specificity. *Dietary Prevention of Recurrence*: While fiber helps symptoms, the idea that specific dietary interventions can prevent long-term recurrence or modify the disease course remains an un-proven hypothesis awaiting prospective validation.

### 8.4. Key Areas for Future Research Include

*Efficacy and Safety of Targeted Therapies*. Prospective studies, including well-designed randomized trials and high-quality observational cohorts, are needed to define the efficacy, safety, and appropriate patient selection for anti-TNF-α therapies in SCAD. While current evidence suggests a potential role for TNF-α–driven inflammation in SCAD pathogenesis, this remains a preliminary hypothesis requiring validation through robust clinical data, long-term safety profiling, and assessment of outcomes such as durability of response and immunogenicity. *Clinical Utility of Emerging Biomarkers*. Biomarkers such as miRNAs, oxidative stress mediators, and microbiome-derived signatures show promise for diagnosis and monitoring, but their clinical utility will depend on standardized assays, rigorous analytical and clinical validation, cross-cohort replication, and demonstration of tangible impact on clinical decision-making, treatment selection, and cost-effectiveness before routine adoption. *Impact of Dietary Interventions on Recurrence*. Although high-fiber diets may offer symptom relief and partial risk reduction in DD, prospective research is needed to clarify the effects of specific dietary interventions on recurrence, quantify the magnitude of risk reduction, and develop evidence-based, long-term prevention guidelines that account for adherence, individual variability, and nutritional adequacy.

### 8.5. A Short Interpretative Model Integrating FC Levels, Radiology, Endoscopy, and Histology

The diagnostic pathway for SCAD typically begins with clinical suspicion in older patients presenting with characteristic symptoms and known DD, followed by initial laboratory testing to exclude infectious etiologies. Non-invasive biomarkers such as FC, together with imaging studies, aid in differentiating SCAD from other forms of colitis. Endoscopic evaluation frequently reveals inflammation localized to the segment between diverticula. Definitive diagnosis is established by histopathologic examination of mucosal biopsies, which demonstrates chronic active colitis confined to the diverticular segment.

## Figures and Tables

**Table 1:** Medication management.

Indications	Pharmacological Management	References
SCAD syndrome	-without pharmacological or biological treatment	[57]
In patients with mild to moderate distal colitis corresponding to diverticular-affected segments.	**5-ASA (orally or via topical enema)** - *Drug Class*: Aminosalicylates (non-immunosuppressive anti-inflammatory agents). - *Mechanism*: 5-ASA acts topically on the colonic mucosa. Key proposed mechanisms include: inhibition of Inflammation, antioxidant activity, immune modulation. - *Evidence Level*: induction of remission, clinical efficacy, maintenance: selective recommendation. - *Key References:* Clinical Review (2024): Clinical Characteristics and Treatment of SCAD. Treatment Guidelines (2021/2026): Segmental Colitis Associated with Diverticulosis (IntechOpen). Pathogenesis & Management Review (2023): Segmental Colitis Associated with Diverticulosis (SCAD)—PMC. Pharmacology Summary: Mesalamine (5-ASA) StatPearls Update (2024).	[58,59]
Diverticulitis patients	**1. Intravenous (IV) Fluids** - *Drug Class*: Isotonic Crystalloids (Supportive care/Rehydration). - *Mechanism*: Restores intravascular volume and maintains electrolyte balance in patients with severe pain or systemic symptoms who are unable to tolerate oral intake (bowel rest). - *Evidence Level*: Standard of care for hospitalized patients with complicated or severe diverticulitis (moderate-quality expert consensus). - *Key References*: AAFP Diagnosis and Management of Acute Diverticulitis. **2. Antibiotics (Intravenous or Oral)** - *Drug Class*: Broad-spectrum Antibiotics (e.g., Nitroimidazoles, Fluoroquinolones, Penicillins). - *Mechanism*: Target aerobic and anaerobic pathogens (e.g., *E. coli*, *B. fragilis*) to resolve infection and prevent complications like abscess or perforation. - *Evidence Level*: Complicated Diverticulitis: Strong recommendation (High-quality evidence). Uncomplicated Diverticulitis: Conditional/Selective recommendation (Low-quality evidence). Recent trials suggest they are not routinely required for immunocompetent patients with mild disease. - *Key References*: AGA Clinical Practice Update (2021/2026). Cochrane Review on Antibiotics for Uncomplicated Diverticulitis (2022). **3. Probiotics** - *Drug Class*: Probiotic Microorganisms (Biologicals/Nutraceuticals). - *Mechanism*: Potential modulation of gut microbiota, inhibition of pathogen adherence, and anti-inflammatory effects through cytokine regulation. - *Evidence Level*: Not recommended for routine use (Low-quality evidence). Current guidelines suggest insufficient evidence for their role in preventing recurrence or symptom relief. - *Key References*: AGA Guidelines on the Role of Probiotics (2020/2026). NCBI Management of Diverticular Disease Evidence Review (2025).	[60,61]
When response to 5-ASA is inadequate or in cases with more extensive disease:	**Locally Active Corticosteroid for SCAD** - *Drug Class*: Second-generation Corticosteroids (Glucocorticoids). - *Mechanism:* High First-Pass Metabolism, Anti-inflammatory Action: - *Evidence Level:* Level III/Grade C (Moderate/Low-quality evidence). Recommendations are primarily based on case series, post hoc analyses, and expert consensus, as large-scale randomized controlled trials (RCTs) specifically for SCAD remain limited as of 2026. - *Key References:* Clinical Studies: Budesonide MMX for Persistent Symptoms in Diverticular Disease (2019/2025). Guideline Context: The Diagnosis, Pathology, and Treatment of Diverticular Colitis (SCAD). Treatment Algorithms: Segmental Colitis Associated with Diverticulosis: SCAD Overview.	[62]
In cases where steroid-sparing strategies are required or if the disease demonstrates chronicity:	**Conventional Immunosuppressants (Azathioprine/6-MP)** - *Drug Class:* Thiopurines (Purine antimetabolites/Immunomodulators). - *Mechanism*: Purine Inhibition, Immune Cell Suppression, Apoptosis Induction, Steroid-Sparing. - *Evidence Level:* Level IV/Grade D (Expert Opinion/Very Low-quality evidence) for SCAD specifically. While high-quality evidence exists for their use in UC (maintenance of remission: moderate/high-certainty), their application in SCAD is extrapolated from IBD guidelines due to the rarity of chronic, refractory SCAD cases. - *Key References:* Guideline Context: Segmental Colitis Associated with Diverticulosis—IntechOpen (2021/2026 update). Treatment Algorithms: Stepwise Treatment Algorithm for SCAD Refractory Cases (PMC). Pharmacology & IBD Standards: Cochrane Review: Thiopurines for Maintenance in UC (2025 Update) and Azathioprine for Steroid-Dependent Colitis (2025). Clinical Review: Segmental Colitis Associated with Diverticulosis (SCAD) Pathology & Treatment.	[63]

## Data Availability

No new data were created or analyzed in this study.
